# Naturally-Occurring Genetic Variants in Human DC-SIGN Increase HIV-1 Capture, Cell-Transfer and Risk of Mother-To-Child Transmission

**DOI:** 10.1371/journal.pone.0040706

**Published:** 2012-07-10

**Authors:** Geneviève Boily-Larouche, Miroslav P. Milev, Lynn S. Zijenah, Annie-Claude Labbé, Djimon M. Zannou, Jean H. Humphrey, Brian J. Ward, Johanne Poudrier, Andrew J. Mouland, Éric A. Cohen, Michel Roger

**Affiliations:** 1 Laboratoire d’Immunogénétique, Centre de Recherche du Centre Hospitalier de l’Université de Montréal (CRCHUM), Montréal, Canada; 2 Département de Microbiologie et Immunologie, Université de Montréal, Montréal, Canada; 3 Department of Medicine, McGill University, The Lady Davis Institute for Medical Research and McGill AIDS Center, Montreal, Canada; 4 Department of Immunology, University of Zimbabwe College of Health Sciences, Harare, Zimbabwe; 5 Département de Microbiologie de l’hôpital Maisonneuve-Rosemont, Montréal, Canada; 6 Centre National Hospitalier Universitaire, Université d’Abomey Calavi, Cotonou, Bénin; 7 Department of International Health, Johns Hopkins Bloomberg School of Public Health, Baltimore, Maryland, United States of America; 8 Research Institute of the McGill University Health Centre, Montreal, Canada; 9 Institut de Recherches Cliniques de Montréal, Montréal, Canada; South Texas Veterans Health Care System and University Health Science Center San Antonio, United States of America

## Abstract

**Background:**

Mother-to-child transmission (MTCT) is the main cause of HIV-1 infection in children worldwide. Dendritic cell–specific ICAM-3 grabbing-nonintegrin (DC-SIGN, also known as CD209) is an HIV-1 receptor that enhances its transmission to T cells and is expressed on placental macrophages.

**Methods and Findings:**

We have investigated the association between DC-SIGN genetic variants and risk of MTCT of HIV-1 among Zimbabwean infants and characterized the impact of the associated mutations on DC-SIGN expression and interaction with HIV-1. DC-SIGN promoter (p-336C and p-201A) and exon 4 (198Q and 242V) variants were all significantly associated with increased risk of intrauterine (IU) HIV-1 infection. Promoter variants decreased DC-SIGN expression both in vitro and in placental CD163^+^ macrophages (Hofbauer cells) of HIV-1 unexposed infants but not of HIV-1 exposed infants. The exon 4 protein-modifying mutations increased HIV-1 capture and transmission to T cells in vitro.

**Conclusion:**

This study provides compelling evidence to support an important role of DC-SIGN in IU HIV-1 infection.

## Introduction

In 2010, UNAIDS estimates that 390,000 children acquired HIV-1-infection worldwide mostly through mother-to-child transmission (MTCT) [1]. Overall transmission rates in the absence of any intervention vary from 12 to 42%. Although antiretroviral therapy (ART) can reduce MTCT to as low as 2% [2], limited access to timely diagnostics and drugs in resource-poor settings blunts the potential impact of this strategy. A better understanding of the mechanisms acting in MTCT of HIV-1 is crucial for the design of interventions other than ART for transmission prevention.

MTCT of HIV-1 can occur during pregnancy (in utero, IU), at delivery (intrapartum, IP) and via breastfeeding (postpartum, PP). HIV-1 can cross the placental barrier in utero either by microtransfusion or by transcytosis across the trophoblast cell layer [2]. IP transmission may occur through direct contact between infant mucosa and HIV-1 infected maternal blood and/or cervico-vaginal secretions [2]. Finally, HIV-1 in breast milk may result in PP infection of the newborn through mucosal exposure [2]. High maternal viral loads in serum and breast milk and low CD4 cell count as well as obstetric factors such as preterm delivery, vaginal delivery, and prolonged membrane rupture have been correlated with increased risk of MTCT of HIV-1 [2], [3].

Genetic variations in HIV-1 co-receptors and determinants of immunity have been shown to influence the outcome of MTCT of HIV-1 [2], [4]. Variants that result in either increased CCR5 expression or a non-functional receptor (32 base-pair deletion variant) influenced risk of vertical transmission [5], [6]. The CCR5 32 base-pair deletion is absent in African populations [7]. Genetic polymorphism of innate immunity determinants such as toll-like receptor 9 and mannose-binding protein also increased the risk of MTCT [8]–[10]. Discordance at the human leucocyte antigen (HLA) class I loci between mother and child or specific HLA alleles also protect against MTCT [11], [12].

Dendritic cell–specific ICAM-3 grabbing-nonintegrin (DC-SIGN, encoded by CD209) is a C-type lectin that binds to many pathogens including HIV-1 [13]. This interaction with HIV-1 leads to viral capture and subsequent transmission to adjacent T cells [14], [15]. DC-SIGN is expressed on the cell surface of myeloid dendritic cells and some macrophage subsets including Hofbauer cells present in the placenta [13], [16]. In the context of HIV-1, DC-SIGN may not only promote trans-infection of T cells but signalling initiated by HIV-1 binding may also influence immune responses and enhance productive infection of the dendritic cells themselves [17]–[19].

Given the presence of DC-SIGN in the placenta and its known interaction with HIV-1, we hypothesized that polymorphism affecting its expression or function might influence the risk of MTCT of HIV-1. Here, we report significant associations between DC-SIGN genetic variants that modulate DC-SIGN expression in placental macrophages, promote HIV-1 capture and transmission to T cells and increase risk of MTCT among Zimbabwean infants.

## Methods

### Subjects

We studied a subgroup of 197 infants born to ART-naive HIV-1-infected mothers recruited in the ZVITAMBO study, which enrolled 14,000 mother-baby pairs between November 1997 and January 2000 in Harare, Zimbabwe [20]. ART prophylaxis for HIV-1-positive antenatal women was not available in the Harare public-sector during ZVITAMBO patient recruitment. The samples were consecutively drawn from two groups: 97 HIV-1-positive mother/HIV-1-positive child pairs and 100 HIV-1-positive mother/HIV-1-negative child pairs. Modes of infant HIV-1 transmission were determined using definitions adapted from Bryson and colleagues [21] and were described elsewhere [22]. Full methods for recruitment, baseline characteristics collection, laboratory procedures have been described elsewhere [20]. MTCT of HIV in the whole ZVITAMBO cohort occurred during the IU (22,9%), IP (48%) and PP periods (29.1%) [20].

### DC-SIGN Haplotypes Reconstruction, htSNPs Selection and Genotyping

Haplotype reconstruction was performed as previously described [23]. Haplotype-tagged single nucleotide polymorphisms (htSNPs) were determined using the HaploBlockFinder software with a minor allele frequency over 5% [24] and numbers were redefined compared to our previous publication [23] for their frequency in the present study population. Ten htSNPs were selected corresponding to the 10 major haplotypes from the 20 SNPs (rs number in Table S1) found in the Zimbabwean population as we previously described [23]. These htSNPs along with the 3 others exon 4 mutations were genotyped in the 197 infants by direct PCR sequencing analysis as previously described [23]. Putative transcription factors binding sites in promoter region were analysed with TESS interface (http//:www.cbil.upenn.edu/tess) using the TRANSFAC database.

### Luciferase Assay

Genomic DNA from homozygous patients with or without mutation was amplified in the promoter region from nucleotide −507 to −1 and cloned between the Bgl II and Hind III multiple cloning sites in the pGL2-Basic vectors (Invitrogen, Canada inc, Burlington, Canada). All recombinants clones were verified by DNA sequencing. Luciferase assay was performed as previously described [22], [25]. Firefly luciferase reporter vector was co-transfected with constitutive expressor of Renilla luciferase, phRL-CMV (Promega, Madison, WI, USA). Firefly luciferase activity was normalized to Renilla luciferase activity.

**Table 1:** Associations between child DC-SIGN haplotypes and intrauterine (IU), intrapartum (IP) and postpartum (PP) HIV-1 transmission.

Child DC-SIGN haplotype^a^	HIV-1 negative		IU infection		IP infection		PP infection
	% (n)	% (n)	OR (95% CI)	% (n)	OR (95% CI)	% (n)	OR (95% CI)
			P value^c^		P value^c^		P value^c^
**H1**							
Absent^b^	80 (159)	84 (86)	1.0	86 (19)	1.0	94 (32)	1.0
Present	20 (39)	16 (16)	0.76 (0.40–1.44)	14 (3)	0.64 (0.18–2.29)	6 (2)	0.25 (0.06–1.11)
			0.444		0.770		0.053
**H2**							
Absent	60 (118)	81 (83)	1.0	82 (18)	1.0	35 (12)	1.0
Present	40 (80)	19 (19)	0.34 (0.19–0.60)	18 (4)	0.33 (0.11–1.01)	65 (22)	0.80 (0.38–1.72)
			0.0002		0.062		0.700
**H3**							
Absent	90 (179)	88 (90)	1.0	82 (18)	1.0	85 (29)	1.0
Present	10 (19)	12 (12)	1.26 (0.58–2.70)	18 (4)	2.10 (0.64–6.83)	15 (5)	1.62 (0.56–4.69)
			0.560		0.260		0.370
**H4**							
Absent	98 (190)	90 (92)	1.0	91 (20)	1.0	91 (31)	1.0
Present	2 (4)	10 (10)	5.27 (1.61–17.3)	9 (2)	4.85 (0.84–28.2)	9 (3)	4.69 (1.00–22.0)
			0.0025		0.110		0.067
**H5**							
Absent	94 (187)	88 (90)	1.0	95 (21)	1.0	88 (30)	1.0
Present	6 (11)	12 (12)	2.27 (0.96–5.33)	5 (1)	0.81 (0.09–6.59)	12 (4)	2.27 (0.68–7.59)
			0.066		1.00		0.250
**H6**							
Absent	91 (180)	80 (82)	1.0	82 (18)	1.0	79 (27)	1.0
Present	9 (18)	20 (20)	2.44 (1.23–4.86)	18 (4)	2.22 (0.68–7.28)	21 (7)	2.59 (0.99–6.79)
			0.0095		0.250		0.070
**H7**							
Absent	93 (185)	95 (97)	1.0	95 (21)	1.0	100 (34)	
Present	7(13)	5 (5)	0.73 (0.25–2.12)	5 (1)	0.67 (0.08–5.45)	0	NA
			0.790		1.00		

CI, Confidence interval; n, number; NA, non applicable, OR, odds ratio.

a Haplotypes found at a frequency above 5% in the study population.

b Absent (referent category for all analyses) vs homozygote + heterozygote (present) for each haplotypes.

c P-value as determined by Fisher’s exact test.

**Table 2:** Associations between child H4 and H6 DC-SIGN htSNPs and intrauterine (IU) HIV-1 transmission.

Child DC-SIGN htSNPs^a^	HIV-1 negative	IU	Crude OR (95% CI)	Adjusted OR (95% CI)
	% (n)	% (n)	P value^b^	P value^c^
				
p-336 T/C				
TT	64 (42)	39 (7)	1.0	1.0
CT/CC	36 (57)	61 (44)	4.63 (1.90–11.3)	4.87 (1.91–12.4)
			0.0004	0.0008
p-201 C/A				
CC	81 (80)	63 (32)	1.0	1.0
CA/AA	19 (19)	37 (19)	2.50 (1.73–5.62)	2.76 (1.20–6.33)
			0.0160	0.0155
R198Q				
GG	81 (80)	57 (28)	1.0	1.0
AG/AA	19 (19)	43 (21)	3.16 (1.48–6.72)	3.32 (1.46–7.52)
			0.0032	0.0038
L242V				
CC	96 (95)	84 (41)	1.0	1.0
GC/GG	4 (4)	16 (8)	4.63 (1.32–16.2)	5.12 (1.36–19.2)
			0.0205	0.0146
				

CI, Confidence interval; htSNPs, haplotype-tagged single nucleotide polymorphisms n, number; NA, non applicable, OR, odds ratio.

a Wild-type (referent category for all analyses) vs homozygote + heterozygote for each htSNPs.

b P-value as determined by Fisher’s exact test.

c Adjusted for the maternal viral load in logistic regression analysis.

### Capture and Transmission Assay

Site-directed mutagenesis was carried out using pcDNA3-DC-SIGN vectors obtained from Drs. S. Pöhlmann, F. Baribaud, F. Kirchhoff and R.W. Doms [26] through the AIDS Research and Reference Reagent Program, NIAID, NIH: pcDNA3-DC-SIGN exon 4 was amplified from genomic DNA of variants carriers and replaced between PspI and EspI (Fermantas, Burlington, Canada) restriction sites. All recombinants clones were verified by DNA sequencing for the presence of mutations of interest and conservation of the coding frame. Stably transfected cell lines were generated from Raji cells (ATCC, Manassas, USA) by nucleofection (Cell line Nucleofector Kit V, Amaxa, Walkersville, USA) and maintained in RPMI 1640 10% FBS containing 1 mg/ml of G418 (Invitrogen). DC-SIGN-expressing cells were sorted (sorter BD ARIA, BD Biosciences, Mississauga, Canada) and limiting dilutions were performed. Cell lines were grown from a single clone. 3×10^5^ cells/well of each Raji transfectants were plated in 96-well plates and pre-incubated with mannan (200 µg/ml, Sigma-Aldrich, St-Louis, USA), DC-SIGN blocking antibody (clone AZN-D1, R&D systems, Minneapolis, USA) (20 µg/ml), matching isotype control or medium for 30 minutes at 4°C before adding 50 ng of p24 equivalent of virus (HIV-1_HxBru-ADA_ or HIV-1_JRCSF_). Cells were incubated 2 h at 37°C and washed with PBS (Invitrogen). Cells were lysed and assayed for p24 Ag using ELISA. In co-culture experiment, Raji transfectants were pulsed as above and 3×10^5^ phytohemagglutinin-L activated human primary CD4^+^ T-lymphocytes (ratio 1∶1) isolated from peripheral blood mononuclear cells were added. Cells were cultivated in RPMI 10% FBS 100 U/ml rIL-2 for 5 days. Supernatants were collected and p24 was measured using ELISA. CD4^+^ T-lymphocytes were isolated from healthy donors and activated as previously described [27]. HIV-1 stocks were generated by transient transfection of HEK293T cells with HxBRU ADA encoding the R5-tropic HIV-1ADA Env [28] or JRCSF proviral construct using the standard calcium-phosphate method. Viral stock was titrated using ELISA p24 Ag (BioChain, Hayward, USA).

**Figure 1:**
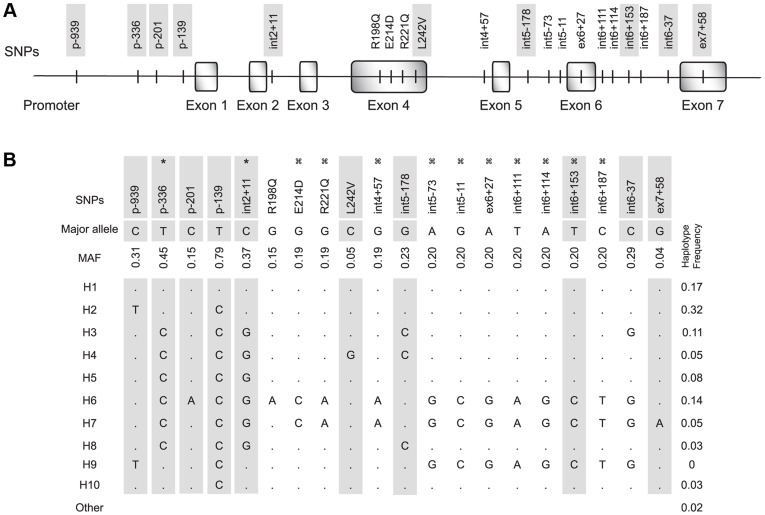
Inferred haplotypes for DC-SIGN in Zimbabwean population. (A) Schematic representation of the location of the 20 single nucleotide polymorphisms (SNPs) selected in the DC-SIGN gene. Minor allele frequency (MAF) >5%. (B) DC-SIGN inferred haplotypes. Frequencies of minor alleles are indicated. Dots referred to the major allele. The 10 haplotype-tagged SNPs are shaded. * and ζ referred to SNPs in complete linkage disequilibrium (D’ = 1).

### Flow Cytometry

DC-SIGN expression was monitored by flow cytometry analysis using FITC-labelled anti-DC-SIGN antibodies clones DCN46 (BD Biosciences, Missisaugua, Canada) and 5D7 (Santa Cruz Biotechnology, Santa Cruz, USA). The cells were also incubated with isotype-matched control antibodies. Flow cytometry was performed using a BD FACS-Scan (BD Biosciences). Full-term placentas were obtained following non-complicated pregnancies and deliveries at Hôpital St-Luc and Hôpital Bethesda in Cotonou, Benin. All infants were delivered vaginally except for two who were delivered by caesarean section and none of the mothers presented with signs of sexually transmitted infections or placental malaria infection. Placentas with signs of inflammation (chorioamnionitis) were excluded. These HIV-1-infected mothers received a combination of three antiviral drugs (full regimen) during pregnancy and delivery. Infants received a full regimen or a single dose nevirapine at delivery and none of them were HIV-infected. A small piece of each placenta was collected and processed within 3 hours following the delivery and washed extensively with PBS to remove blood and maternal cells. Mononuclear cells were mechanically isolated from placental tissue using a Medimachine (BD Biosciences) and purified on Histopaque gradient (Sigma-Aldrich, Oakville, Canada). Placental mononuclear cells were cryopreserved until flow cytometry analysis. Cells from 4 wild-type (WT) p-336T/p-201C and 11 homozygote or heterozygote p-336C/p-201A infants for the promoter variants born to HIV-1-negative mothers were analysed. Placental cells from 3 WT and 6 homozygote or heterozygote infants born to HIV-1-positive mothers were also analysed. Placental cells were analysed by flow cytometry using CD3-, CD19- (eBioscineces, San Diego, USA), and CD56-PerCPCy5.5 (BD Biosciences), CD14-Alexa700 (BioLegend, San Diego, USA), CD163-APC (R&D System, Minneapolis, USA), DC-SIGN-FITC, CD68-PE and HLA-DR-PECy7 (BD Biosciences) antibodies and isotype-matched controls. Dead cells stained with Live/Death Aqua (Invitrogen) and lineage cells stained with CD3-, CD19- and CD56-PerCPCy5.5 antibodies were excluded. Placental macrophages were initially gated for CD14 expression and high granularity. Geometric mean fluorescence intensity (MFI) was calculated in DC-SIGN^+^CD163^+^ and DC-SIGN^+^CD163^−^ subsets to assess the level of DC-SIGN expression and their level of maturation. Change geometric MFI represents the difference between specific marker expression and its FMO (fluorescence minus one). Flow cytometry was performed using a BD LSR-Fortessa (BD Biosciences).

**Figure 2:**
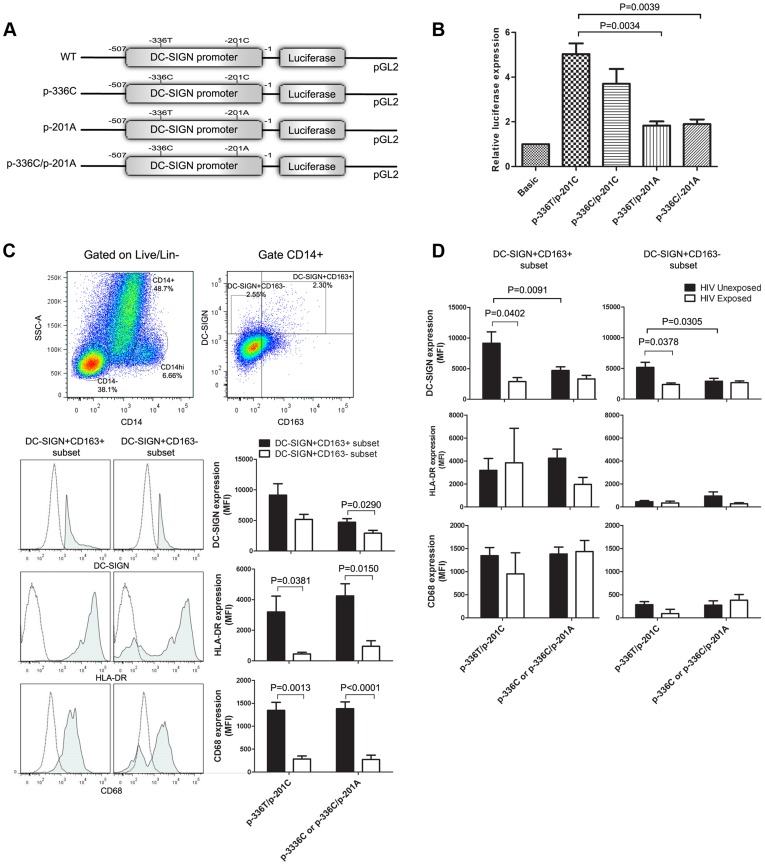
DC-SIGN promoter variants reduced transcriptional activity *in vitro* and reduced DC-SIGN expression in placental macrophages. (A, B) Transcriptional activity *in vitro* (A) Schematic representation of reporter gene constructs corresponding to the DC-SIGN promoter region from positions −507 to −1 with or without promoter variants −336C and −201A. (B) Relative luciferase expression from pGL2-Basic, the parental vector without a promoter. Expression of the DC-SIGN promoter constructs was calculated relative to this value. Results are mean ± S.E.M. values of three independent experiments performed in triplicates and differences in relative luciferase expression between variants and wild-type were examined with Student’s t test. (C) Hofbauer-like cells were analysed by flow cytometry to measure DC-SIGN expression in infants bearing or not promoter variants. Dead cells and Lin^+^ (CD3; CD19; CD56) cells were excluded and subsets were identified for their side scatter (SSC-A) properties and their level of CD14 expression. Placental macrophages were selected for high granularity and CD14 expression (CD14^+^ subset). DC-SIGN was expressed on CD163^+^ and CD163^−^ subsets. Dot plots and flow cytometry histograms are representative experiments of all patients. Mean fluorescence intensity (MFI) of DC-SIGN, HLA-DR and CD68 was compared between both subsets for infants bearing or not promoter variants and born from HIV-1-negative mothers (p-336T/p-201C group n = 4; p-336C or p-336C/p-201A group n = 11). (D) DC-SIGN, HLA-DR and CD68 expression was compared in CD163+ and CD163− subsets from infants bearing or not promoter variants and born from HIV-1-negative mothers (HIV-1 Unexposed; p-336T/p-201C group n = 4; p-336C or p-336C/p-201A group n = 11) or from HIV-1-positive mothers (HIV-1 Exposed; p-336T/p-201C group n = 3; p-336C or p-336C/p-201A group n = 6). Results in C and D are mean ± S.E.M. values of MFI and difference between subsets or variants was calculated with Student’s t test.

### Statistical Analysis

Statistical analysis was performed using GraphPad PRISM 5.0 for Windows (GraphPad Software inc. San Diego, CA). In order to assess the association between each of the DC-SIGN hapotype (Table 1) or htSNP (Table 2) alleles with MTCT of HIV-1, those subjects who were heterozygous and homozygous for the haplotype or htSNP alleles were compared separately with subjects who tested negatively for that allele (reference category). The association between each of the putative haplotype or htSNP alleles and risks of MTCT of HIV-1 was investigated using crude and adjusted multivariate logistic regression to derive odds ratio (OR) and 95% confidence interval (CI) as estimates of relative risks. Specifically, the models were adjusted for the maternal viral load. The analyses were restricted to those haplotypes found at a frequency above 5% in the study population (Table 1) or to those SNPs found only in H4 or H6 (Table 2). Differences in frequencies of haplotypes and htSNPs were compared between groups using Fisher’s exact test. All SNPs were in Hardy-Weinberg Equilibrium [23]. For luciferase DC-SIGN/HLA-DR/CD68 expression, capture and transmission assays comparisons between WT and variants were assessed with the unpaired two-tailed Student’s t test.

### Ethics Statement

Written informed consent was obtained from all mothers who participated in the study. The study was approved by The Medical Research Council of Zimbabwe, The Medicines Control Authority of Zimbabwe, The Johns Hopkins Bloomberg School of Public Health Committee on Human Research, and the CHUM and Montreal General Hospital Ethics Committees. Full-term placental tissues were obtained following written informed consent in accordance with the Comité National Provisoire d’Éthique de la Recherche en Santé in Cotonou (Benin) and the CHUM Research Ethics Committee. Peripheral blood samples were obtained from healthy, HIV-1-seronegative adult donors who gave written informed consent in accordance with the Declaration of Helsinki under research protocols approved by the research ethics review board of the Institut de Recherches Cliniques de Montréal.

**Figure 3:**
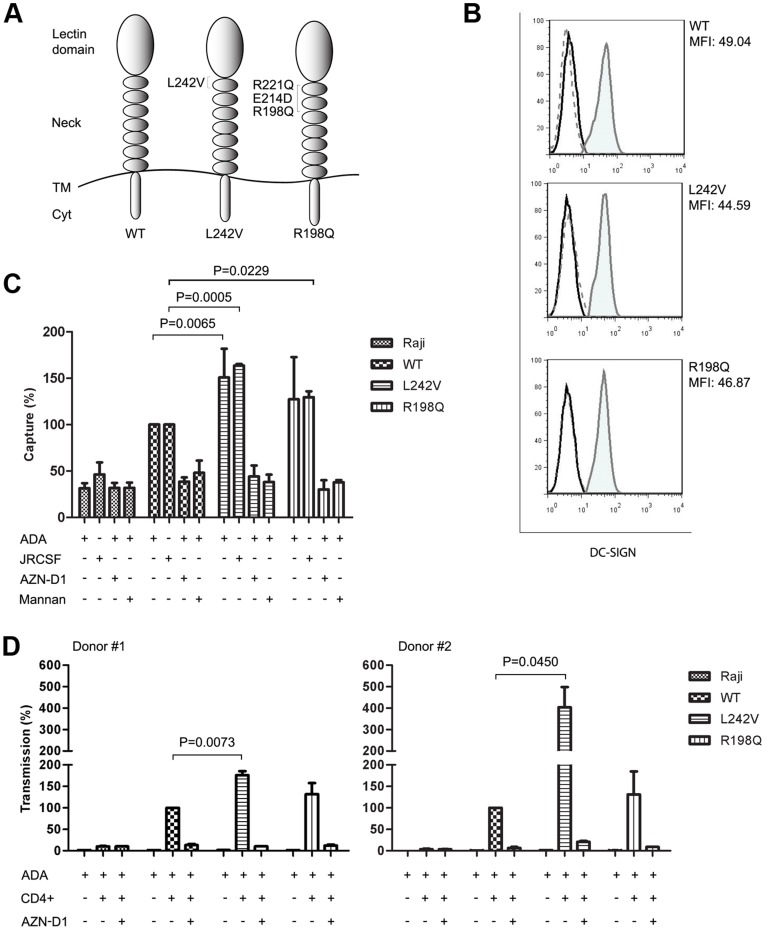
DC-SIGN neck variants enhance HIV-1 capture and transmission. (A) Schematic representation of DC-SIGN constructs representing DC-SIGN neck variants stably expressed in the Raji cell line. (TM; transmembrane domain, Cyt; cytoplasmic domain). (B) Raji-transfectants were selected for similar DC-SIGN cell-surface expression by flow cytometry. Cells stained with anti-DC-SIGN (DCN46) (filled grey histogram) or isotypic control (dashed grey line) are shown. Parental Raji cells are represented by the black line. Antibody titration was achieved at the same dilution for all cell lines using two DC-SIGN monoclonal antibodies (clones DCN46 and 5D7) that recognized different epitopes. (C) HIV-1 capture by DC-SIGN variants. Raji-transfectants were incubated with HIV-1_HXBru-ADA_ (ADA) or HIV-1_JRCSF_ (JRCSF) for 2 h at 37°C, extensively washed and lysed. Cell-associated p24 Ag was measured by ELISA. Where indicated, cells were pre-incubated with anti-DC-SIGN (AZN-D1) or with mannan to inhibit DC-SIGN interaction with HIV-1. HIV-1 capture is shown relative to WT (WT = 100%). (D) HIV-1 transfer to T lymphocytes by DC-SIGN variants. Raji-transfectants were pulsed as in (C) and subsequently co-cultivated with activated human primary CD4^+^T lymphocytes from two donors for 5 days. Virus release into the supernatant was measured by ELISA p24. Where indicated, cells were pre-incubated with AZN-D1. HIV-1 transmission is shown relative to WT (WT = 100%). Results are mean ± SD of duplicates for each donor (D) or three independent experiments (C). Student’s t test was used to calculate differences in % capture and transmission among Raji DC-SIGN transfectants L242V, R198Q and WT.

## Results

### DC-SIGN Genetic Variants are Associated with Increased Risk of IU HIV-1 Infection

We carried out an association study of DC-SIGN polymorphism in 197 infants born to ART-naive HIV-1-infected mothers recruited in Harare, Zimbabwe [20]. Among them, 97 were HIV-1-infected and 100 were uninfected. Of the 97 HIV-1-infected infants, 57 were infected IU, 11 IP, and 17 PP. Timing of infection could not be determined for 12 HIV-1-infected infants as specimens were not available at some time points. Baseline characteristics of mothers and infants were reported previously [22]. Briefly, maternal age and CD4^+^ T cell count, child sex, mode of delivery, duration of membrane rupture and gestational age were similar among all groups. Maternal viral load >29 000 copies/ml was associated with increased risk of both IU and PP HIV-1 transmission, OR: 3.64, 95% CI: 1.82–7.31, P = 0.0002 and OR: 4.45, 95% CI: 1.50–13.2, P = 0.0045, respectively.

Ten htSNPs from the 20 SNPs (Figure 1A) corresponding to the 10 major DC-SIGN haplotypes (Figure 1B) previously described among Zimbabweans [23], were genotyped in the study samples. Haplotypes with frequencies above 5% in the study population were analysed for their potential association with MTCT of HIV-1. Infants carrying H4 or H6 haplotypes had increased risk of IU HIV-1-infection, whereas H2 haplotype carriers were less likely to be infected during pregnancy compared to infant noncarriers (Table 1). None of the haplotypes were significantly associated with altered risks of IP or PP infections. The H4 and H6 hapotypes remained significantly associated with IU HIV-1 infection (OR: 4.98, 95% CI: 1.32–18.8, P = 0.0168 and OR: 2.93, 95% CI: 1.27–6.76, P = 0.0113, respectively) after adjustment was made for maternal viral load. H2 haplotype remained significantly associated with protection against IU infection after adjustment for maternal viral load (OR: 0.23, 95% CI: 0.10–0.51, P = 0.0003). To identify the causal SNPs associated with increased IU transmission of HIV-1, we determined the association between IU HIV-1 infection and each of H4 and H6 signature SNPs. Promoter p-201A (rs11465366) and exon 4 198Q (rs41374747) variants are found exclusively in H6 while exon 4 242V (rs11465380) variant tag H4 (Figure 1B). Both H4 and H6 haplotypes harbour promoter variant p-336C (rs4804803) that is known to influence DC-SIGN promoter activity [25] and increased risk of HIV-1 parenteral infection [29]. These variants were all associated with increased risk of IU HIV-1 infection after adjustment for maternal viral load (Table 2). In a step-wise logistic regression analysis including all DC-SIGN associated SNPs and maternal viral load, DC-SIGN 242V variant (OR: 4.87, 95% CI: 1.19–19.9, P = 0.0261) and maternal viral load (OR: 3.30, 95% CI: 1.48–7.37, P = 0.0033) remained independent predictors of HIV-1 IU acquisition. Maternal DC-SIGN haplotypes were not associated with MTCT of HIV-1 (Table S2).

We have previously investigated the association between DC-SIGN-related (DC-SIGNR, encoded by *CD209L*) genetic variants and MTCT of HIV-1 in the same subset of infants [22]. DC-SIGNR is a DC-SIGN homologue expressed at the cell-surface of endothelial cells of placental capillaries [30]. DC-SIGNR promoter p-198A and intron 2 180A variants were significantly associated with increased risk of MTCT. When adjustment was made for all the significant DC-SIGN and DC-SIGNR associations in logistic regression analysis, DC-SIGN exon 4 242V (OR: 5.03, 95% CI: 1.18–21.4, P = 0.0275) and DC-SIGNR intron 2 180A (OR: 6.93, 1.51–31.7, P = 0.0119) variants remained associated with increased risk of IU transmission, suggesting that DC-SIGN and DC-SIGNR are independent predictors of IU of HIV-1 among Zimbabweans.

### Promoter Variants Reduce Transcriptional Activity *in vitro* and DC-SIGN Expression in Hofbauer Cells

We next investigated the impact of the HIV-1 associated promoter variants on both DC-SIGN transcriptional activity *in vitro* and expression in fetal macrophages (Hofbauer cells). Variant p-336C decreased the transcriptional activity of Sp1 binding site [25], [31]. Transcription factor binding site analysis predicted that variant p-201A would create a c-myc binding site. To test the effect of these promoter variants on transcription, we transiently transfected HeLa cells with a luciferase reporter gene under the control of DC-SIGN promoter region -507 to -1 containing AP-1, Sp1, Ets-1 and NF-KB transcription factors that are essential for promoter activity [32] and harbouring promoter WT p-336T/p-201C or variant p-336C/p-201A sequences (Figure 2A). As previously reported [25], [31], the luciferase activity of the p-336C/p-201C variant construct was lower than that of WT p-336T/p-201C (Figure 2B) but the decreased did not reach significance in our assay. The p-201A variant either alone or in combination with p-336C significantly reduced DC-SIGN transcriptional activity *in vitro* (Ratio p-336T/p-201C/p-336C/p-201A = 3.13, P = 0.0039). Uninfected infants harboured more frequently H1 and H2 haplotypes reaching significance for H2 (Table 1). H2 carries two promoter variants, p-939T and p-139C, that differ from WT H1 haplotype (Figure 1B). The promoter variants p-939T and p-139C did not show any influence on DC-SIGN transcriptional activity *in vitro* (Figure S1).

However, HeLa cells derived from cervical carcinoma might not represent the best model to study the impact of promoter variants on DC-SIGN expression in macrophages. To address this issue, we further determine the net impact of susceptibility-associated promoter mutations on DC-SIGN expression by measuring total DC-SIGN protein expression in Hofbauer cells. These cells are found within the chorionic villi beneath the syncytiotrophoblast layer at the maternal-fetal interface [33]. Term placentas contain a distinct population of Hofbauer cells that co-express DC-SIGN, CD163, CD14, CD68 and HLA-DR, a phenotype similar to alternatively activated macrophages (M2) known for their immunosuppressive properties [16], [33], [34]. Hofbauer cells were analysed by flow cytometry after isolation of mononuclear cells from term placentas of promoter WT p-336T/p-201C and variants p-336C/p-201A carriers. CD14^+^ cells of high granularity that were negative for T, B and NK cell markers (CD3, CD19 and CD56) were identified as Hofbauer cells (CD14^+^ population) and two subsets of DC-SIGN^+^ cells were observed (CD163^+^ and CD163^−^, Figure 2C). CD163^+^ cells expressed significantly higher levels of DC-SIGN, HLA-DR and CD68 compared to CD163^−^ cells (Figure 2C). We then compared levels of DC-SIGN expression in CD163^+^ and CD163^−^ cells between infants carrying or not carrying promoter variants and born from HIV-1-negative or HIV-1-positive mothers (Figure 2D). In infants born to HIV-1-negative mothers, levels of DC-SIGN expression were reduced 1.9-fold (P = 0.0091) in CD163^+^ cells and 1.8-fold (P = 0.0305) in CD163^−^ cells from infants carrying the promoter variants compared to infants harbouring the WT promoter sequence. Interestingly, DC-SIGN expression varied according to the mothers’ HIV-1 status. In infants harbouring the WT sequence, levels of DC-SIGN expression were reduced 3.2-fold (P = 0.0402) by CD163^+^ cells and 2.2-fold (P = 0.0378) by CD163^−^ cells in infants born from HIV-1-positive mothers compared to infants born from HIV-1-negative mothers. However, it remained unchanged in infants carrying the promoter variants. Hence, p-336C and p-201A altered DC-SIGN expression in placental Hofbauer cells and but their impact vary according maternal HIV-1 status.

### Protein-modifying Variants Increase Viral Capture and Transfer to T cells

DC-SIGN molecules on the cell surface enhance HIV-1 infection by capturing virions and transmitting them to CD4^+^ T-lymphocytes [14], [15]. The neck region, encoded by exon 4, is important for efficient binding to HIV-1 [35]. We hypothesized that the exon 4 protein-modifying variants associated with IU HIV-1 infection could affect the interaction between DC-SIGN and HIV-1. To assess viral capture, exon 4 from the DC-SIGN expression vector was replaced by exon 4 from infants carrying WT, 242V (designated as L242V) or 198Q, 214D and 221Q (designated as R198Q) variants (Figure 3A). Raji cells do not express endogenous DC-SIGN and allowed us to investigate the net impact of exon 4 mutations on DC-SIGN HIV-1 affinity. Raji cells (Figure 3B) were stably transfected and cell lines grown from a single clone. Since viral capture is influenced by cell-surface expression of DC-SIGN [35], we selected cell lines with similar baseline DC-SIGN surface expression (Figure 3B). The stable Raji transfectants were pulsed with equal amount of R5 tropic HIV-1_HXBRU-ADA_ or HIV-1_JRCSF_ strains extensively washed to remove the unbound virus, and then lysed. The parental Raji cells were used as controls. The number of virions used was not saturating since capture increased in a dose-dependent manner (Figure S2A). Interestingly, DC-SIGN L242V and R198Q variants were more efficient at capturing viral particles than WT (Figure 3C). HIV-1 capture on the Raji transfectants was stable over time (Figure S2B) and dependent on DC-SIGN interaction since the capture was reduced to background levels following incubation with DC-SIGN antibody (AZN-D1) or mannan (Figure 3C). Similar results were obtained when cells were pulsed with HIV-1_JRCSF_ strain (Figure S2C). To investigate whether DC-SIGN exon 4 mutations could also enhance cell transmission of HIV-1, we co-cultivated activated primary human CD4^+^ T lymphocytes with HIV-1 pulsed Raji transfectants. Transmission was quantified by measuring HIV-1 p24 in the supernatants after 5 days. The DC-SIGN variants significantly increased viral transmission to CD4^+^ T-lymphocytes (Figure 3D). Transmission was dependent on DC-SIGN expression since Raji cells or transfectants pre-incubated with DC-SIGN antibody failed to transmit HIV-1 to CD4^+^ T lymphocytes. Moreover, cell infection was not due to viral particles shed into the supernatant since virus was undetectable in the absence of CD4^+^ T lymphocytes (Figure 3D). Thus, the DC-SIGN neck region variants associated with IU HIV-1 infection enhanced both the capture of HIV-1 by DC-SIGN and its subsequent transmission to the CD4^+^ T lymphocytes.

## Discussion

*In vitro* studies have shown that the interaction between DC-SIGN and HIV-1 can enhance short-term viral transfer to other susceptible cell types such as T lymphocytes [14], [15], [36]. Based on these findings, a Trojan horse model has been proposed whereby HIV-1 may subvert DC-SIGN-expressing submucosal dendritic cells to promote dissemination from the periphery to the lymphoid tissues [13]. To date, relatively few studies have assessed the potential impact of DC-SIGN polymorphism in adult HIV-1 infection and the findings have not been consistent. While some [29], [37], [38] have found a significant association, others have not [39]–[41]. Little is currently known about the mechanisms underlying HIV-1 passage across the placenta. We and others [16], [33], [34] have shown that DC-SIGN is expressed by placental Hofbauer cells. Moreover, the identification of natural and functional DC-SIGN genetic variants associated with an increased risk of IU HIV-1 infection further support the implication of DC-SIGN in HIV-1 dissemination across the placenta. DC-SIGN polymorphism was not associated with IP and PP HIV-1 infections. However, the relatively small number of subjects analysed in the IP (n = 11) and PP (n = 17) groups may have limited the power of the present study to detect any association and therefore we cannot rule out the possibility that DC-SIGN could also contribute to IP and PP transmission of HIV-1.

DC-SIGN promoter p-336C and exon 4 242V variants are observed on the H4 haplotype while p-336C, p-201A and 198Q variants are found on the H6 haplotype. Thus exon 4 variants are always transmitted with one or both promoter variants. These variants were all associated with IU HIV-1 infection and yet the promoter variants reduced DC-SIGN expression in Hofbauer cells whereas the exon 4 mutations enhanced capture and transmission of HIV-1 to CD4^+^ T lymphocytes. Compensatory mutations frequently evolve to dampen the effect of other mutations. Since the DC-SIGN gene has been under strong evolutionary pressure to conserve its function [42] it is not surprising that mutations increasing the affinity of DC-SIGN for pathogens have appeared that can compensate for mutations that reduce its expression. Interestingly, HIV-1 itself can also affect DC-SIGN expression. Indeed, HIV- or antibody-stimulated DC-SIGN signalling in monocytes-derived dendritic cells (MDDC) reduced DC-SIGN expression and prevented cell maturation [19], [43]. In infants harbouring the WT sequence, levels of DC-SIGN expression were significantly lower in infants born from HIV-1-positive mothers than those born from HIV-1-negative mothers (Figure 2D). However, the impact of HIV-1 was negligible or not noticeable in infants carrying the promoter variants since baseline DC-SIGN expression levels were already low in these subjects. Although we cannot exclude that ART may also modulate DC-SIGN expression, it is reasonable to believe that HIV affects DC-SIGN expression in the tissue since *in vitro* experiments support this hypothesis [19], [43]. In the present study, Zimbabwean infants were born from ART-naive HIV-1-positive mothers. Given the fact that they were all exposed to HIV during their intra-uterine life, they may have harboured similar levels of DC-SIGN expression (Figure 2D). Hence, the positive association observed between IU HIV-1 infection and DC-SIGN H4 and H6 haplotypes may thus result from exon 4 protein-modifying mutations found within these haplotypes that enhanced capture of HIV-1 by Hofbauer cells within the chorionic villi in close proximity to maternal infected cells and facilitate short-term transfer of the virus to the infant’s T lymphocytes [16]. On the other hand, we cannot rule out the possibility that DC-SIGN variants might promote HIV-1 infection of Hofbauer cells and subsequently IU transmission of HIV-1. Hofbauer cells express both the HIV-1 CD4 receptor and the CCR5 co-receptor [16], [44] and HIV-1 genomic materials have been detected in placental macrophages [45]. Kumar *et al* observed compartmentalized HIV-1 replication within the placenta during IU transmission [46] and proposed that viral selection during IU transmission could be the manifestation of HIV-1 placental adaptation to the unique repertoire of cellular targets and increased adherence to C-type lectins which further support the implication of DC-SIGN in IU transmission of HIV-1. However, the net impact of this phenomenon on MTCT of HIV-1 remains to be determined since it has also been shown that placental macrophages can restrict HIV-1 replication [47].

In addition to the enhancement of HIV-1 capture and transmission to target cells, DC-SIGN genetic variants may also contribute to a local immunological environment that promotes viral replication and dissemination of HIV-1 across the placenta [17], [19]. HIV-1 or HIV-1-derived products activated fetal macrophages and T lymphocytes and promoted the establishment of a productive infection within the placenta [46], [48]–[50]. In response to dengue infection, MDCCs from DC-SIGN p-336CT heterozygous individuals produced higher levels of pro-inflammatory factors such as TNF-alpha, IL-12 and IP-10 than those from WT p-336TT homozygotes [51]. Moreover, TNF-alpha enhanced HIV-1 replication and transcytosis within the placenta and TNF-alpha level correlated with the amount of HIV-1 transcripts [52]–[54]. It is tempting to speculate that infants harbouring the p-336TT genotype, such as H1 and H2 carriers, may produce less TNF-alpha to reduce or thwart HIV-1 replication in the placenta.

In this study, we demonstrate for the first time, the impact of DC-SIGN natural polymorphisms on its expression in placental cells and interaction with HIV-1 and provide compelling evidence to support an important role of DC-SIGN in IU HIV-1 infection. These findings raise the possibility that similar mechanisms may operate with other human pathogens known to interact with DC-SIGN and warrant further investigation.

## Supporting Information

Table S1 **Description of** ***DC-SIGN*** **polymorphisms.**
(DOCX)

Table S2 **Associations between maternal DC-SIGN haplotypes and intrauterine (IU), intrapartum (IP) and postpartum (PP) HIV-1 transmission. CI, Confidence interval; n, number; NA, non applicable, OR, odds ratio.** ^a^Haplotypes found at a frequency above 5% in the study population. Absent (referent category for all analyses) vs homozygote + heterozygote for each haplotypes. ^b^P-value as determined by by Fisher’s exact test.
(DOCX)

Figure S1 **Effect of DC-SIGN promoter variants on transcriptional activity.** (a) Schematic representation of reporter gene constructs corresponding to the DC-SIGN promoter region from positions −1055 to −1 with or without promoter variants −939 and −139. (b) Relative luciferase expression from pGL2-Basic, the parental vector without a promoter. Expression of the DC-SIGN promoter constructs was calculated relative to the value of pGL2-Basic, which was arbitrarily set as 1. Data are mean ± SD values of 3 independent experiments performed in triplicates and there were no significant differences in the relative expression between variants and wild-type (WT) as determined with Student’s *t* test.
(DOCX)

Figure S2 HIV-1 capture by Raji transfectants (a) Dose-dependent HIV-1 capture by Raji transfectants. Raji and Raji-H1 transfectants were incubated with 25, 50 and 100 ng of p24-equivalent of HIV-1_HXBru-ADA_ for 2 h at 37°C, washed with cold PBS 1X and lysed in 0,5% Triton X-100. Cell-associated p24 contents were measured by ELISA. (b) Residual cell-associated HIV-1 over time. 3×10^5^ Raji-transfectants were exposed to 150 ng of p24-equivalent of HIV-1_HXBru-ADA_ for 2 h at 37°C, washed and incubated in fresh medium at 37°C for different time points (0, 1 h, 2 h, 4 h and 18 h). Cell-associated p24-contents were measured by ELISA after lysis in 0,5% Triton X-100. (c) Capture assay with HIV-1_JR-CSF_. 3×10^5^ cells were incubated with 50 ng of p24-equivalent of HIV-1_JR-CSF_ for 2 h at 37°C, washed extensively with cold PBS 1X and lysed in 0,5% Triton X-100. Cell-associated p24 contents were measured by ELISA. Where indicated, cells were pre-incubated 30 min at 4°C with 20 µg/ml of anti-DC-SIGN (AZND1) or with mannan (200 µg/ml) to inhibit DC-SIGN interaction with HIV-1 before pulsing with HIV-1_JR-CSF_. HIV-1 capture is shown relative to wild-type (WT = 100%). Data are mean ± SD of 2 independent experiments performed in duplicates. Student’s *t* test was used to calculate differences in % capture between the Raji DC-SIGN transfectants L242V, R198Q and WT.
(DOCX)
