# Supplementary material for: Naturally-Occurring Genetic Variants in Human DC-SIGN Increase HIV-1 Capture, Cell-Transfer and Risk of Mother-To-Child Transmission
Source: PLoS One. 2012 Jul 10;7(7):e40706. doi: 10.1371/journal.pone.0040706 (PMC3393705; doi:10.1371/journal.pone.0040706)
Supplement: Table S1 — Description of DC-SIGN polymorphisms. (DOCX) [file pone.0040706.s001.docx]

**Table S1** Description of *DC-SIGN* polymorphisms

| SNPs | Chromosome | Position on | NCBI | Position on cDNA | Nucleotide change | Amino Acid change |
| --- | --- | --- | --- | --- | --- | --- |
|  | # | NT_077812.2 | rs number | NM_021155 |  |  |
| p-939 | 19 | 417,332 | rs735240 | 5' UTR | C/T |  |
| p-336 | 19 | 416,729 | rs4804803 | promoter | T/C |  |
| p-201 | 19 | 416,594 | rs11465366 | promoter | C/A |  |
| p-139 | 19 | 416,532 | rs2287886 | promoter | T/C |  |
| int2+11 | 19 | 416,177 | rs7252229 | intron 2 | C/G |  |
| R198Q | 19 | 414,555 | rs41374747 | exon 4 | G/A | R198Q |
| E214D | 19 | 414,506 | rs11465377 | exon 4 | G/C | E214D |
| R221Q | 19 | 414,486 | rs41335247 | exon 4 | G/A | R221Q |
| L242V | 19 | 414,424 | rs11465380 | exon 4 | C/G | L242V |
| int4-57 | 19 | 414,031 | rs11465383 | intron 4 | G/A |  |
| int5-178 | 19 | 413,279 | rs8105483 | intron 5 | G/C |  |
| int5-73 | 19 | 413,174 | rs61058460 | intron 5 | A/G |  |
| int5-11 | 19 | 413,112 | rs57668221 | intron 5 | G/C |  |
| ex6+27 | 19 | 413,075 | rs17159887 | exon 6 | A/G |  |
| int6+111 | 19 | 412,876 | ss158145668 | intron 6 | T/A |  |
| int6+114 | 19 | 412,875 | ss158145669 | intron 6 | A/G |  |
| int6+153 | 19 | 412,836 | rs73493975 | intron 6 | T/C |  |
| int6+187 | 19 | 412,802 | rs73493973 | intron 6 | C/T |  |
| int6-37 | 19 | 412,159 | rs11465391 | intron 6 | C/G |  |
| ex7+58 | 19 | 412,065 | rs35545365 | exon 7 | G/A |  |
