# Supplementary material for: Naturally-Occurring Genetic Variants in Human DC-SIGN Increase HIV-1 Capture, Cell-Transfer and Risk of Mother-To-Child Transmission
Source: PLoS One. 2012 Jul 10;7(7):e40706. doi: 10.1371/journal.pone.0040706 (PMC3393705; doi:10.1371/journal.pone.0040706)
Supplement: Table S2 — Associations between maternal DC-SIGN haplotypes and intrauterine (IU), intrapartum (IP) and postpartum (PP) HIV-1 transmission. CI, Confidence interval; n, number; NA, non applicable, OR, odds ratio. aHaplotypes found at a frequency above 5% in the study population. Absent (referent category for all analyses) vs homozygote + heterozygote for each haplotypes. bP-value as determined by by Fisher’s exact test. (DOCX) [file pone.0040706.s002.docx]

**Table S2** Associations between maternal DC-SIGN haplotypes and intrauterine (IU), intrapartum (IP) and postpartum (PP) HIV-1 transmission.

| Maternal DC-SIGN haplotypes^a^ | No Transmission | IU transmission | | IP transmission | | PP transmission | |
| --- | --- | --- | --- | --- | --- | --- | --- |
|  | % (n) | % (n) | OR (95% CI) | %(n) | OR (95% CI) | % (n) | OR (95% CI) |
|  |  |  | P value^b^ |  | P value^b^ |  | P value^b^ |
|  |  |  |  |  |  |  |  |
| **H1**  Absent | 78 (154) | 81 (83) | 1.0 | 82 (22) | 1.0 | 91 (31) | 1.0 |
| Present | 22 (43) | 19 (19) | 1.22 (0.67-2.23) | 18 (4) | 1.26 (0.40-3.91) | 9 (3) | 2.89 (0.84-9.90) |
|  |  |  | 0.52 |  | 1.0 |  | 0.10 |
| **H2**  Absent | 66 (130) | 68 (69) | 1.0 | 77 (17) | 1.0 | 56 (19) | 1.0 |
| Present | 34 (67) | 32 (33) | 1.08 (0.65-1.79) | 23 (5) | 1.75 (0.62-4.96) | 44 (15) | 0.65 (0.31-1.37) |
|  |  |  | 0.77 |  | 0.20 |  | 0.26 |
| **H3**  Absent | 90 (178) | 90 (92) | 1.0 | 82 (18) | 1.0 | 85 (29) | 1.0 |
| Present | 10 (19) | 10 (10) | 0.98 (0.44-2.20) | 18 (4) | 0.48 (0.15-1.57) | 15 (5) | 0.62 (0.21-1.79) |
|  |  |  | 0.96 |  | 0.26 |  | 0.37 |
| **H4**  Absent | 95 (188) | 93 (95) | 1.0 | 91 (20) | 1.0 | 88 (30) | 1.0 |
| Present | 5 (9) | 7 (7) | 0.65 (0.23-1.80) | 9 (2) | 0.48 (0.10-2.37) | 12 (4) | 0.36 (0.10-1.24) |
|  |  |  | 0.40 |  | 0.30 |  | 0.11 |
| **H5**  Absent | 95 (188) | 92 (94) | 1.0 | 95 (21) | 1.0 | 100 (34) |  |
| Present | 5 (9) | 8 (8) | 0.56 (0.21-1.51) | 5 (1) | 1.01 (0.12-8.34) | 0 | NA |
|  |  |  | 0.25 |  | 1.0 |  |  |
| **H6**  Absent | 87 (172) | 84 (86) | 1.0 | 86 (19) | 1.0 | 85 (29) | 1.0 |
| Present | 13 (25) | 16 (16) | 0.78 (0.40-1.54) | 14 (3) | 0.92 (0.25-3.34) | 15 (5) | 0.84 (0.30-2.38) |
|  |  |  | 0.48 |  | 1.0 |  | 0.75 |
| **H7**  Absent | 94 (186) | 96 (98) | 1.0 | 100 (22) |  | 100 (34) |  |
| Present | 6 (11) | 4 (4) | 1.45 (0.45-4.67) | 0 | NA | 0 | NA |
|  |  |  | 0.78 |  |  |  |  |
