# Supplementary figures and images for: Naturally-Occurring Genetic Variants in Human DC-SIGN Increase HIV-1 Capture, Cell-Transfer and Risk of Mother-To-Child Transmission
Source: PLoS One. 2012 Jul 10;7(7):e40706. doi: 10.1371/journal.pone.0040706 (PMC3393705; doi:10.1371/journal.pone.0040706)

**Figure S1**


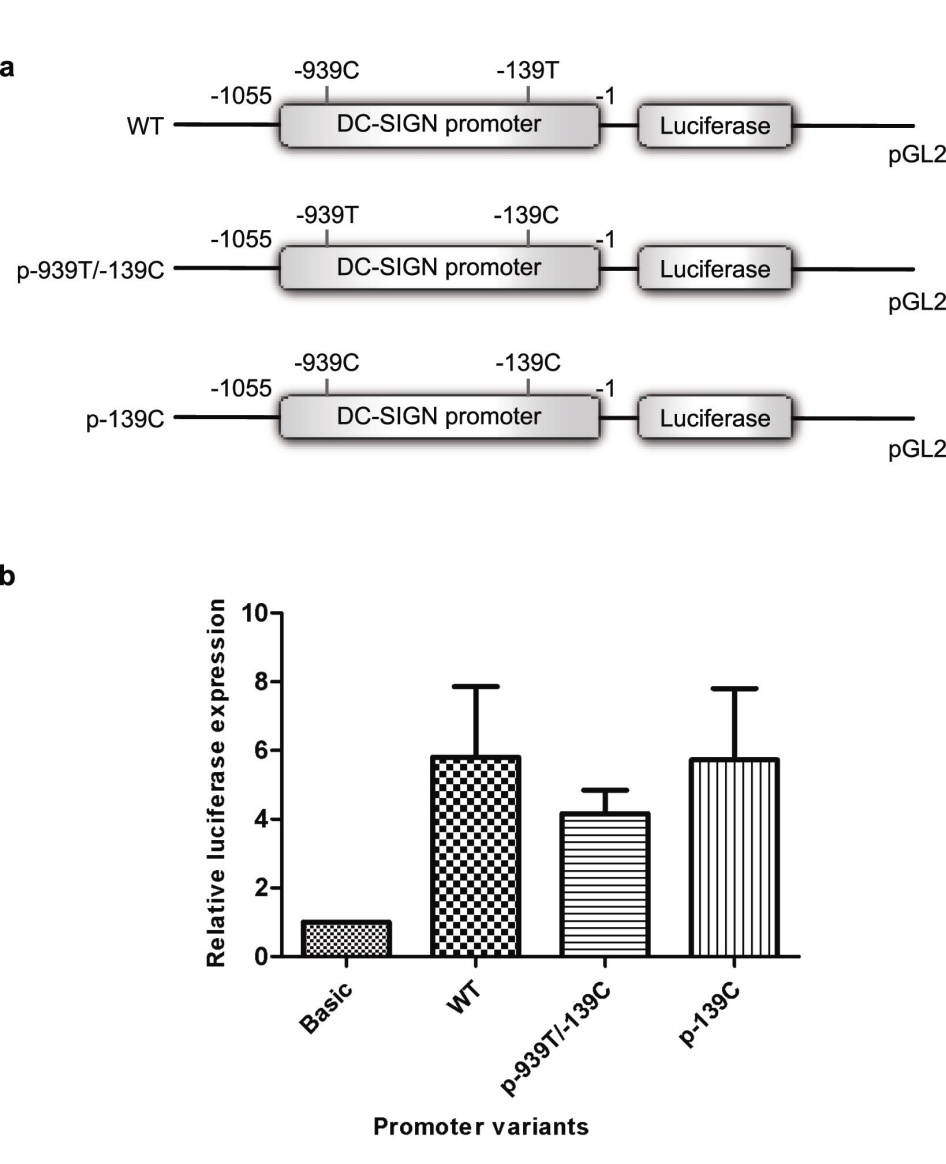

Supplement: Figure S1 — Effect of DC-SIGN promoter variants on transcriptional activity. (a) Schematic representation of reporter gene constructs corresponding to the DC-SIGN promoter region from positions −1055 to −1 with or without promoter variants −939 and −139. (b) Relative luciferase expression from pGL2-Basic, the parental vector without a promoter. Expression of the DC-SIGN promoter constructs was calculated relative to the value of pGL2-Basic, which was arbitrarily set as 1. Data are mean ± SD values of 3 independent experiments performed in triplicates and there were no significant differences in the relative expression between variants and wild-type (WT) as determined with Student’s t test. (DOCX) [file pone.0040706.s003.docx]

**Figure S2**

**
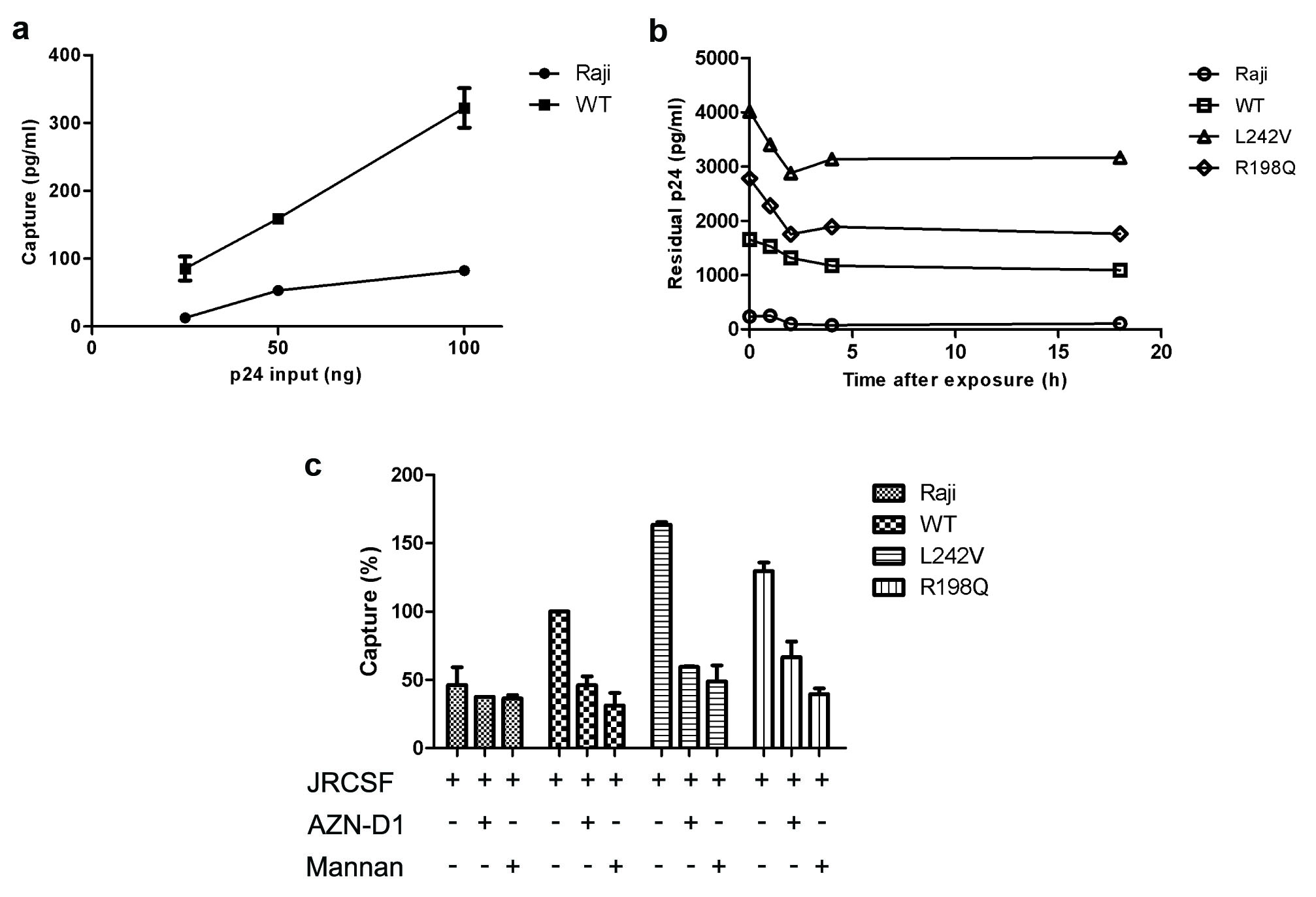
**

Supplement: Figure S2 — HIV-1 capture by Raji transfectants (a) Dose-dependent HIV-1 capture by Raji transfectants. Raji and Raji-H1 transfectants were incubated with 25, 50 and 100 ng of p24-equivalent of HIV-1HXBru-ADA for 2 h at 37°C, washed with cold PBS 1X and lysed in 0,5% Triton X-100. Cell-associated p24 contents were measured by ELISA. (b) Residual cell-associated HIV-1 over time. 3×10^5^ Raji-transfectants were exposed to 150 ng of p24-equivalent of HIV-1HXBru-ADA for 2 h at 37°C, washed and incubated in fresh medium at 37°C for different time points (0, 1 h, 2 h, 4 h and 18 h). Cell-associated p24-contents were measured by ELISA after lysis in 0,5% Triton X-100. (c) Capture assay with HIV-1JR-CSF. 3×10^5^ cells were incubated with 50 ng of p24-equivalent of HIV-1JR-CSF for 2 h at 37°C, washed extensively with cold PBS 1X and lysed in 0,5% Triton X-100. Cell-associated p24 contents were measured by ELISA. Where indicated, cells were pre-incubated 30 min at 4°C with 20 µg/ml of anti-DC-SIGN (AZND1) or with mannan (200 µg/ml) to inhibit DC-SIGN interaction with HIV-1 before pulsing with HIV-1JR-CSF. HIV-1 capture is shown relative to wild-type (WT = 100%). Data are mean ± SD of 2 independent experiments performed in duplicates. Student’s t test was used to calculate differences in % capture between the Raji DC-SIGN transfectants L242V, R198Q and WT. (DOCX) [file pone.0040706.s004.docx]
